# Inelastic X-ray scattering with 0.75 meV resolution at 25.7 keV using a temperature-gradient analyzer

**DOI:** 10.1107/S1600577514021006

**Published:** 2015-01-01

**Authors:** Daisuke Ishikawa, David S. Ellis, Hiroshi Uchiyama, Alfred Q. R. Baron

**Affiliations:** a Materials Dynamics Laboratory, RIKEN SPring-8 Center, 1-1-1 Kouto, Sayo, Hyogo 679-5148, Japan; b Research and Utilization Division, Japan Synchrotron Radiation Research Institute (JASRI), 1-1-1 Kouto, Sayo, Hyogo 679-5198, Japan

**Keywords:** inelastic X-ray scattering, high-resolution analyzer, X-ray optics

## Abstract

The use of temperature-gradient analyzers for non-resonant high-resolution inelastic X-ray scattering is investigated. The gradient compensates for geometrical broadening of the energy resolution by adjusting the lattice spacing of the analyzer crystal. Applying a ∼12 mK temperature gradient across a 9.5 cm analyzer, resolutions of 0.75 (2) meV FWHM at 25.7 keV for Si(13 13 13) and 1.25 (2) meV at 21.7 keV for Si(11 11 11) were measured, while retaining large (250 mm) clearance between the sample position and detector, and reasonable (9.3 mrad × 8.8 mrad) analyzer acceptance. The temperature control and stability are discussed.

## Abstract 2

Temperature-gradient spherical analyzers allow us to improve the resolution of meV-scale inelastic X-ray scattering.

## 1. Introduction   

Non-resonant high-resolution inelastic X-ray scattering (IXS) with ∼1 meV resolution is a spectroscopic technique that has been widely used to study atomic dynamics, including phonons in crystals and collective dynamics in disordered materials. With the advent of intense and highly collimated X-ray beams from synchrotron light sources, much effort has been made to develop instrumentation for IXS spectrometers (Dorner & Peisl, 1983 ▸; Burkel, 1991 ▸; Sette *et al.*, 1998 ▸; Baron *et al.*, 2000 ▸; Sinn *et al.*, 2001 ▸).

A key component in presently operating IXS spectrometers is the spherical analyzer (Fujii *et al.*, 1982 ▸; Masciovecchio *et al.*, 1996*a* ▸,*b* ▸; Verbeni *et al.*, 2005 ▸; Said *et al.*, 2011 ▸), and it is, in fact, usually the analyzer performance that limits the spectrometer energy resolution. Therefore, methods of improving energy resolution of the analyzers are of interest, especially if this is possible without reducing analyzer efficiency (*e.g.* Huotari *et al.*, 2005 ▸, 2006 ▸; Ishikawa & Baron, 2010 ▸). In this paper we show that the temperature-gradient analyzer proposed by Ishikawa & Baron (2010 ▸) can be used to provide 0.75 ± 0.02 meV resolution at the Si(13 13 13) reflection, whereas an analyzer without such a gradient is estimated to provide at best 0.81 meV in theory, and observed to provide not better than 0.9 meV resolution in practice. This validates the temperature-gradient concept for high resolution. In addition, we explore using such a gradient at the Si(11 11 11) reflection and show that it can gain practical improvement in resolution to 1.25 meV, although, for the (11 11 11) reflection, this level of resolution is also theoretically possible with a uniform-temperature crystal.

## 2. Basic concepts for temperature-gradient analyzers   

The essential idea for a temperature-gradient analyzer is to use a thermal gradient to tailor the *d*-spacing of a crystal to compensate for the ‘demagnification’ aberration. This can be useful when the experimental geometry forces the spectrometer to operate without the detector in a conventional Roland geometry, something that becomes increasingly necessary as resolution is improved and spherical analyzers must operate close to backscattering. Ishikawa & Baron (2010 ▸) provide an in-depth discussion of how temperature-gradient analyzers operate. Here we briefly discuss the concept again, but focus on the high (∼meV) resolution case.

### 2.1. Spectrometer geometry   

The high-resolution spectrometer at BL43LXU is designed for ∼meV energy resolution using photon energies *E* = 21.7, 23.7, 25.7 keV [for Si(*nnn*) reflections, *n* = 11, 12, 13]. The monochromator uses a single backscattering reflection with θ_B_ = 89.98°, while the analyzers are ‘diced’ spheres with a radius of curvature *R* = 9.8 m. Of the two geometries discussed by Ishikawa & Baron (2010 ▸), here we use the ‘off-Rowland’ geometry because it minimizes the required size of the detector, reducing noise, and allowing operation closer to backscattering which gives better resolution.

**Figure 1:**
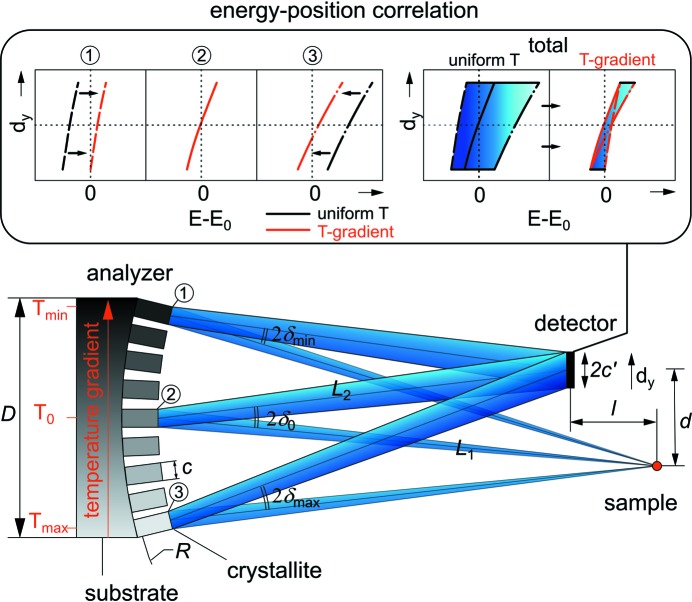
Schematic of the IXS sample–analyzer–detector geometry (side view). Here, *R*, *L*_1_, *L*_2_ 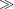 *l* 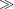 *d*. The analyzer crystals are positioned to focus in the detector. The energy–position (detector vertical position) correlation (*E* − *E*_0_ *versus* *d*_*y*_) of crystallites (1)–(3) and all crystallites effects (total) are also illustrated.

**Figure 2:**
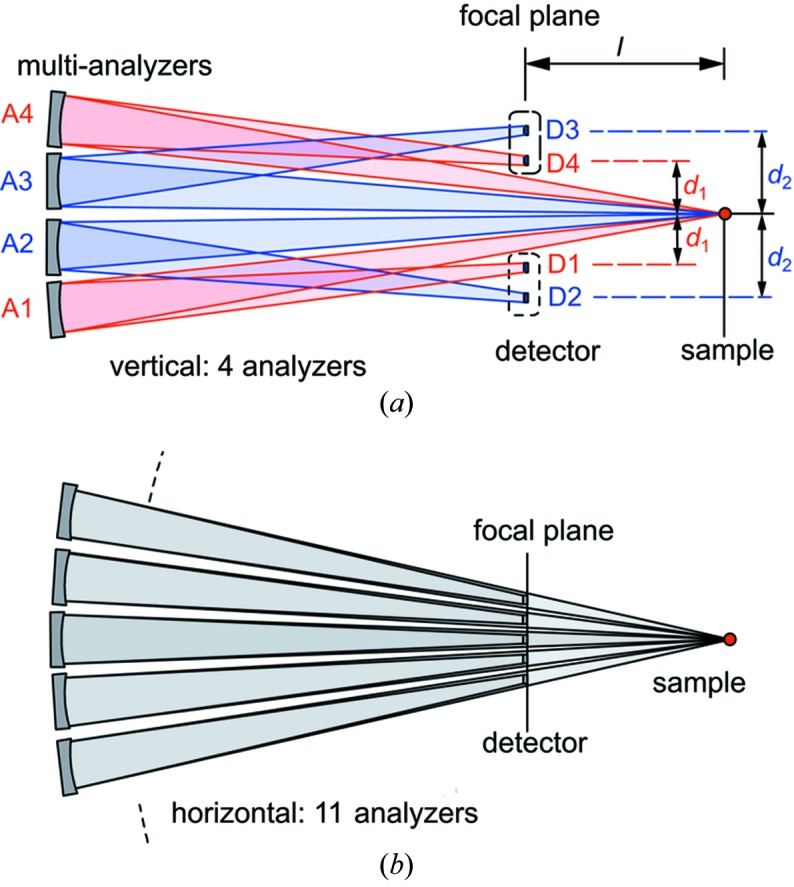
Schematic view of the multi-element analyzer/detector focusing geometry at BL43LXU (not to scale). (*a*) Side view, (*b*) view from above. The signals from each analyzer are collected by individual detectors.

For a spherical analyzer, focusing in the detector requires 2/*R* = 1/*L*_1_ + 1/*L*_2_ where *L*_1_ is the sample–analyzer distance, *L*_2_ the analyzer–detector distance, *l* = *L*_1_ − *L*_2_ is the sample–detector horizontal offset (here, *R* ≃ *L*_1_ ≃ *L*_2_, δ_0_ 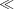 1, δ_0_ ≡ π/2 − θ_B_) (see Fig. 1 ▸). Taking the analyzer radius of curvature *R* = 9800 mm and clearance at sample *l* = 250 mm, then *L*_1_ = 9927 and *L*_2_ = 9679 mm.^1^ Taking the analyzer (vertical) angular acceptance to be Ω = *D*/*L*_1_ = 8.8 mrad, the minimum detector offset in this geometry is given as *d*_min_ = Ω*l*/2 + *c*′ = 3 mm. Here, *c*′ = *c*(1 + *M*)/2 ≃ 0.86, where the analyzer pixel size *c* ≃ 0.87 mm and magnification *M* = *L*_2_/*L*_1_ = 0.975. In order to accommodate four rows of analyzers [as is useful for measuring transverse dispersion; see Baron *et al.* (2008 ▸)], two rows of detectors are used above and below the scattered beam, as shown in Fig. 2(*a*) ▸. This means that the spectrometer has two different offsets, *d*, for the detectors, with *d* = 4 mm for the first and fourth rows of analyzers and *d* = 10 mm for the second and third rows of analyzers. Detector elements have a size of *p* = 2 mm that is sufficient to accept the focused beam size of 2*c*′ ≃ 1.7 mm, and are arranged on a 3.03 mm pitch. Note that this off-Rowland geometry, as discussed by Ishikawa & Baron (2010 ▸), generally requires a non-linear (quadratic) temperature-gradient correction; however, a linear gradient is sufficient in this case due to large, 10 m, arm length and small solid angle Ω ≃ 10 mrad at near-backscattering δ_0_ < 0.5 mrad.

### 2.2. Analyzer crystals   

Analyzers were fabricated on rectangular substrates [100 (H) mm × 95 (V) mm × 30 (t) mm] to facilitate creating a one-directional temperature gradient, as discussed in detail below. Single-crystal silicon was used as a substrate material due to its high thermal conductivity, which both promotes uniform temperature (Masciovecchio *et al.*, 1996*b* ▸) and makes it relatively easy to control the small gradients (∼0.01 K/10 cm) needed for operation. Wafers were cut from a high-purity single-crystalline silicon ingot (resistivity 2–4 kΩ cm) and then diced leaving a 0.2 mm backwall. The wafers were etched in KOH solution, and then the diced side was bonded onto the spherical substrate (*R* = 9800 ± 15 mm) by gold diffusion, before removing the backwall by polishing and etching in acid. The accuracy of the bonded crystallite alignment is typically <17 µrad (r.m.s.) error from ideal curvature on the spherical substrate (Miwa, 2002 ▸). The final crystallites have typical dimensions of 0.87 mm × 0.87 mm × 4.9 mm on a 1.0 mm pitch on the 92 (H) mm × 87 (V) mm active analyzer surface. 5 mm-thick crystallites were used to improve the tails of the resolution function (Said *et al.*, 2011 ▸). To reduce multi-beam effects at the Si(11 11 11) reflection, the (111)-normal wafers were cut with (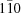) 45° inclined from the (111) scattering plane.

### 2.3. Creating the temperature gradient   

**Figure 3:**
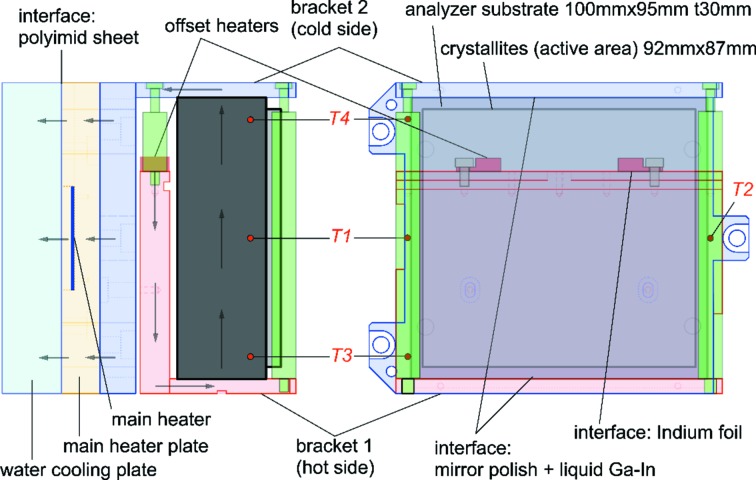
Rectangular analyzer and its holder for a one-directional temperature gradient. *T*_1_–*T*_4_ indicate positions of thermistors used for feedback. The arrows indicate thermal flow under steady-state conditions.

The gradient is created by using the analyzer as one component in a thermal circuit. The required heat flow through the circuit, for a desired temperature gradient Δ*T*/*h*, may be estimated as *Q* = λ*S*Δ*T*/*h*, where, λ is the thermal conductivity, Δ*T* the temperature difference of the two surfaces, *S* the heat transfer area and *h* the heat transfer distance. Taking λ = 156 W m^−1^ K^−1^ [single-crystalline silicon at *T* = 300 K (Glassbrenner & Slack, 1964 ▸)], *S* = 30 mm × 100 mm, *h* = 95 mm and Δ*T*/*h* = 11.9 mK/95 mm (= 10 mK/80 mm), the required power is *Q* = 58.5 mW. To accomplish this, the analyzer is mounted between upper and lower L-shaped angle brackets made of Ni-coated oxygen-free high-conductivity (OFHC) copper. One of the brackets is heated and insulated from the other, as seen in Fig. 3 ▸. A 1 inch × 1 inch film heater (78.4 Ω, Minco HK5163) is used as a main heater for base temperature control, and two power resistors (1.5 W maximum, each 100 Ω, in parallel, Alpha Electronics PDY100R00A) are used to generate the gradient. The assembly is mounted on a water-cooled plate. Heating points are located some distance away from the silicon to improve thermal homogeneity perpendicular to the gradient direction. The assembly is mounted in-vacuum (pressure < 5 Pa) to thermally isolate the system.

The contact between the crystal and the two L-brackets requires care. To improve thermal contact, the surfaces were mirror finished and parallel to <0.1 mrad. To promote temperature uniformity and reduce thermal contact resistance, several materials were tried, including indium foil, silver paste, thermal grease and InGa eutectic. The best results were obtained with a small amount of InGa eutectic.

Temperatures were measured using glass-encapsulated thermistors with a ∼2 mm-diameter bead, 10 kΩ resistance (at 298 K) and four-wire readout, with four temperature sensors per gradient analyzer (Fig. 3 ▸). The sensors *T*_1_ and *T*_2_ were mounted near the center of the substrate at each side, and sensors *T*_3_ and *T*_4_ were attached 80 mm apart each from other. Sensors *T*_1_ and *T*_2_ were for monitoring the temperature of the center of the analyzer, *T*_0_ [≡ (*T*_1_ + *T*_2_)/2], while the sensors *T*_3_ and *T*_4_ were for monitoring the temperature gradient in the vertical direction, Δ*T*_g_ (≡ *T*_3_ − *T*_4_). *T*_0_ and Δ*T*_g_ were used as the feedback parameters for control of the main heater and the offset heater, respectively.

### 2.4. Temperature control and stability   

**Figure 4:**
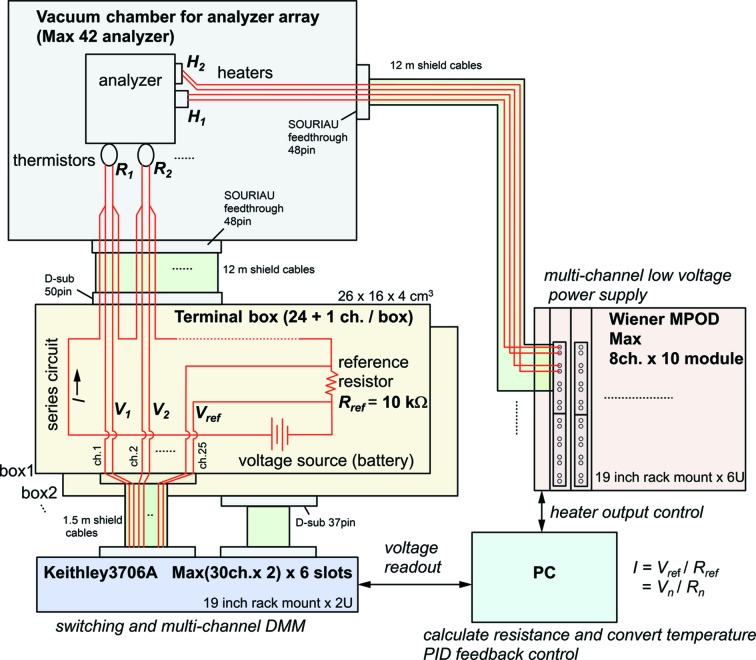
Schematic of the compact multi-channel temperature control system.

The temperature control system was built in-house in order to keep it both compact (see Fig. 4 ▸) and precise over many channels operated in parallel. Standard 1.5 V batteries (AA-cell) are used as ultralow-noise power supplies for the thermistors. The voltage drop across each thermistor is then compared with that across a precision resistor using a switching digital multimeter (Keithley 3706A) with plug-in multiplexers cards (model 3720, 7.5 digit). Typically 24 thermistors are used in parallel with a given reference resistor. The heater power was controlled using a multi-channel low-voltage power supply (W-IE-NE-R MPOD crate) with low-voltage modules type MPV-8060 (16-bit resolution, maximum 50 V). The temperature parameters *T*_0_ and Δ*T*_g_ were controlled by a PID feedback program based on Bechhoefer (2005 ▸), which has online monitoring and logging. The feedback loop takes about 9 s per iteration to scan 72 temperature channels.

**Figure 5:**
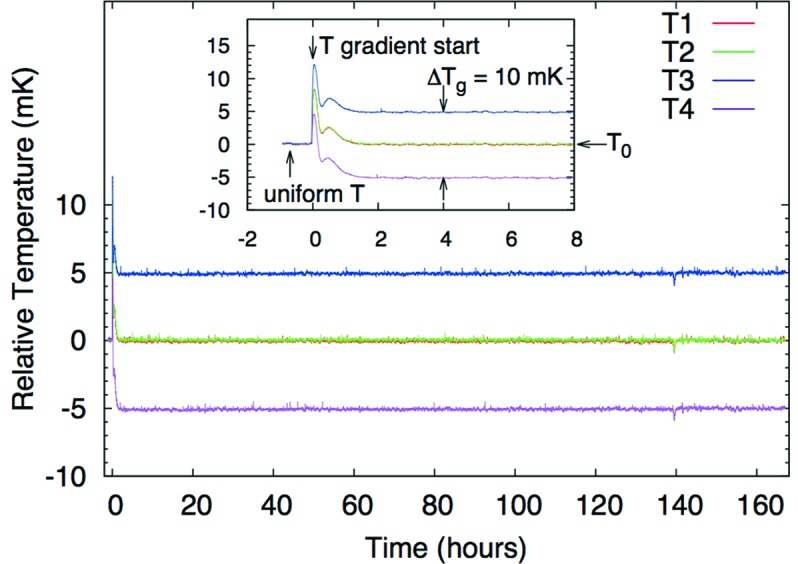
Temperature stability of the analyzer over one week. The temperature gradient is set to Δ*T*_g_ = 10 mK/80 mm. *T*_1_–*T*_4_ are sensors positions as indicated in Fig. 3 ▸. The inset shows the transient when establishing the temperature gradient (Δ*T*_g_ = 0 to 10 mK/80 mm).

The temperatures must be stable to better than ±0.3 mK^2^ for this system to work properly over the ∼day, or longer, time scale of IXS measurements. In order to determine the PID feedback parameters, the open-loop thermal frequency response of the analyzer over long time scales was measured. Bode plots were made from 0.2 to 6 mHz, and the amplitude and phase open-loop response of the analyzer were fit to polynomials, which were used to construct a model of the analyzer’s thermal system in frequency space. Fig. 5 ▸ shows the temperature stability over a period of seven days, with temperature gradient Δ*T*_g_ = 10 mK/80 mm. The stability was within ±0.3 mK (≤0.08 mK r.m.s.) for all temperature sensors. During the initial adjustment phase, it took a few hours for the system to reach steady-state condition (*e.g.* from Δ*T*_g_ = 0 to 10 mK; see inset in Fig. 5 ▸) after a change. Measurement of the energy resolution multiple times over a 30 day period confirmed the stability of the setup. The analyzer array is now operating with 72 channels of readout and 40 channels for control.

## 3. Results and discussion   

Tests were performed at BL43LXU at SPring-8 in Japan (Baron, 2010 ▸). Two different geometries, *d* = 4 and 10 mm (Fig. 2*a* ▸), and two different photon energies, 21.7 and 25.7 keV, were tested. The bandwidth of the X-rays from a segmented undulator source was reduced by a liquid-nitrogen-cooled high-heat-load double-crystal Si(111) monochromator followed by a pair of Si(400) offset crystals. A high-resolution 1.25° grazing-incidence backscattering monochromator using the Si(13 13 13) or Si(11 11 11) reflection was then used to reduce the bandwidth to below 1 meV. The focal spot size at the sample was 40 µm (H) × 45 µm (V) after focusing by an elliptically bent cylindrical mirror. The resolution was measured using a 2 mm-thick polymethyl-methacrylate (PMMA) sample with the analyzer placed at the structure-factor maximum. The full analyzer surface was illuminated, corresponding to momentum resolutions of Δ*Q* ≃ 1.25 nm^−1^ (full-width) at 25.7 keV and Δ*Q* ≃ 1.05 nm^−1^ (full-width) at 21.7 keV X-rays. The incident energy was scanned by changing the temperature of the backscattering monochromator with slope of 5 mK min^−1^ for 25.7 keV and 10 mK min^−1^ for 21.7 keV X-rays, while the analyzer temperature was kept constant. The energy scale was calibrated as described by Fukui *et al.* (2008 ▸).

### 3.1. Si(13 13 13), *d* = 4 and 10 mm   

**Figure 6:**
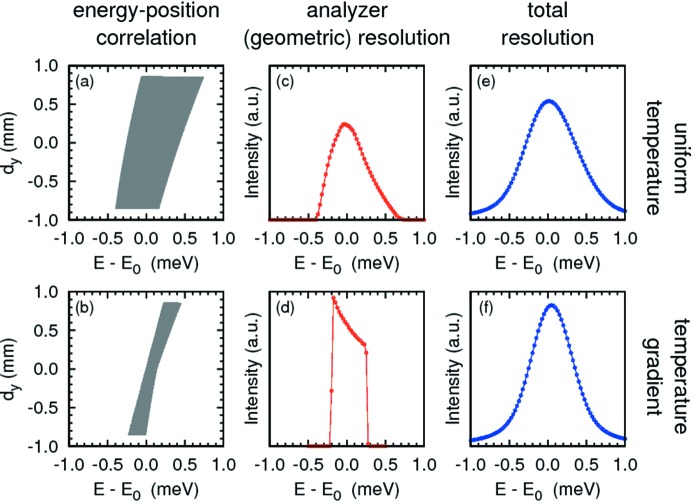
Simulated results for case 1 (*E* = 25.7 keV, *d* = 4 mm) with and without the temperature gradient. (*a*, *b*) Energy transfer *versus* detector vertical position. (*c*, *d*) Geometric resolution function. (*e*, *f*) Total resolution function convolved with the intrinsic response of the of Si(13 13 13) reflections.

**Figure 7:**
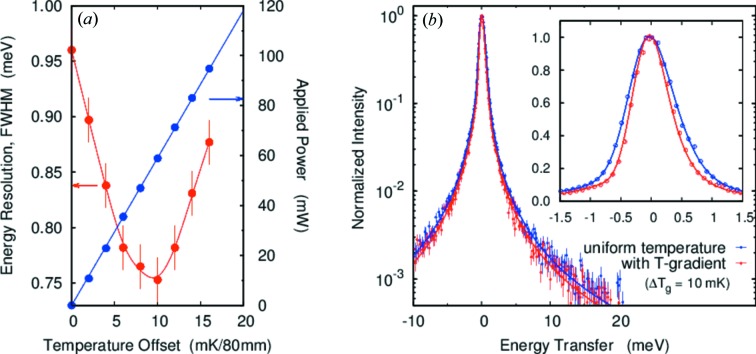
Resolution with the temperature gradient for *E* = 25.7 keV, *d* = 4 mm (case 1). (*a*) Energy resolution as a function of temperature gradient. The curve is to guide the eye. (*b*) Experimental resolution with best-case gradient (Δ*T*_g_ = 10 mK/80 mm) and uniform temperature. The solid lines are fitted curves. The solid angle is 9.3 (H) mrad × 8.8 (V) mrad.

The best resolution was obtained using a temperature gradient at the Si(13 13 13) reflection, *E* = 25.7 keV and *d* = 4 mm. Fig. 6 ▸ shows the calculated energy–position correlation in the detector for a uniform temperature (top) and temperature gradient (bottom). The geometric contribution (Table 1 ▸, case 1) is calculated to be Δ*E*_geom_ = 0.57 meV (Fig. 6*c* ▸) for uniform temperature and 0.43 meV (Fig. 6*d* ▸) for linear temperature gradient. The measured resolution as a function of the applied gradient is shown in Fig. 7 ▸, with the resolution improving from 0.96 meV without a gradient to 0.75 meV at the optimum gradient Δ*T*_g_ = 10 mK/80 mm (58.7 mW) and then becoming worse as the optimum gradient is exceeded. The values of 0.75 and 0.96 meV are both slightly larger than our ray-tracing results of 0.65 and 0.81 meV, respectively, possibly resulting from residual non-uniformity in the lattice spacing over the analyzer surface. However, the value of 0.75 meV is, to the best of our knowledge, the narrowest resolution achieved with a spherical analyzer with ∼10 mrad acceptance. The gradient also allows us to improve the resolution beyond the best value calculated without the gradient. It is also notable that the width of the tails on the resolution function are reduced (Table 3) by the gradient and that there are no changes in the integrated intensities to a level of 2%.

**Figure 8:**
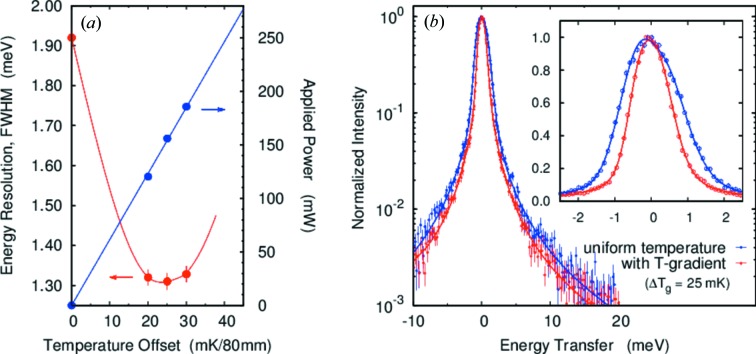
Resolution with the temperature gradient for *E* = 25.7 keV and *d* = 10 mm (case 2). See caption for Fig. 7 ▸. The best-case gradient is Δ*T*_g_ = 25 mK/80 mm.

For the *d* = 10 mm case the geometrical contribution to the resolution is about 2.5 times larger than for *d* = 4 mm due to the larger deviation from backscattering, (Δ*E*/*E*)_geom2_ ≡ tanδ_0_Δδ (Table 1 ▸, case 2). A larger gradient is then expected. Figs. 8(*a*) and 8(*b*) ▸ show the temperature gradient and resolution as a function of temperature gradient in case 2 (*d* = 10 mm). The energy resolution has a minimum at around Δ*T*_g_ = 25 mK/80 mm (155.9 mW), which is roughly consistent with estimated value of Δ*T*_g_ = 20 mK/80 mm (118 mW) as noted in Table 2 ▸. The experimental energy resolution was improved 32% from Δ*E*_tot_ = 1.92 to 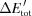 = 1.31 meV (FWHM). The observed minimum resolution 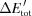 = 1.31 meV is in reasonable agreement with our calculated limit 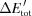 = 1.21 meV (FWHM). Note that, again, the tails on the resolution function are reduced compared with operation without the gradient (Table 3 ▸, cases 1 and 2) and no changes are observed in integrated intensities.

### 3.2. Si(11 11 11), *d* = 4 and 10 mm   

**Figure 9:**
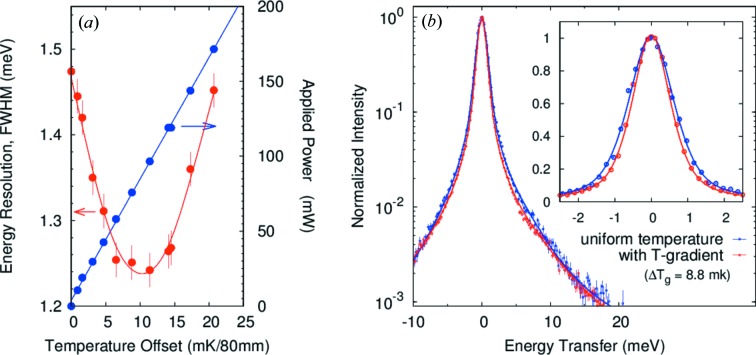
Resolution with the temperature gradient for *E* = 21.7 keV and *d* = 4 mm (case 3). See caption for Fig. 7 ▸. The best-case gradient is Δ*T*_g_ = 8.8 mK/80 mm.

**Figure 10:**
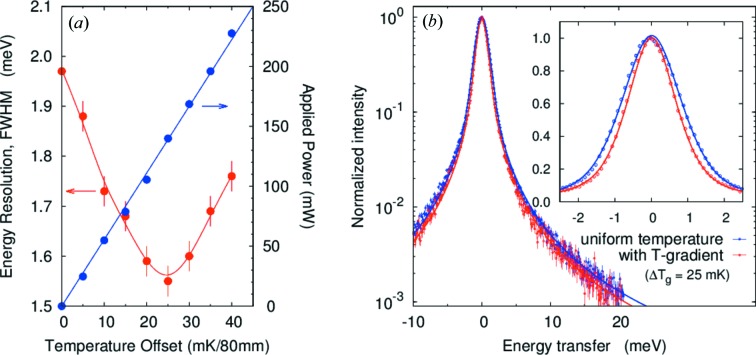
Resolution with the temperature gradient for *E* = 21.7 keV and *d* = 10 mm (case 4). See caption for Fig. 7 ▸. The best-case gradient is Δ*T*_g_ = 25 mK/80 mm.

Operation at the Si(11 11 11) reflection is interesting because the relaxed resolution, compared with the Si(13 13 13) reflection, allows an increase in count rates, although it is still desirable to obtain the best possible resolution. For the *d* = 4 mm geometry, the calculated contribution of the demagnification aberration is small enough that the gradient does not make that big a difference: 1.27 meV without the gradient becomes 1.21 meV at the optimal gradient. However, we find that the observed resolution improved from 1.48 meV to 1.25 meV by adding the gradient (Fig. 9*b* ▸). While this suggests the presence of some residual gradient even when the gradient heater output was zero, it also suggests that practically some improvements can be made. For the case of *d* = 10 mm, when the analyzers are further from backscattering, the calculated improvement (1.72 to 1.45 meV) is more substantial, and we find that the best-case measured resolution is 1.55 meV (Fig. 10 ▸).

## 4. Conclusion   

We have shown that careful application of a temperature gradient allows us to improve the resolution of ∼meV-scale-resolution spherical analyzers. We show a significant, ∼22%, improvement in resolution, with a best case of 0.75 meV (FWHM) using 25.7 keV X-rays, without loss of intensity. The gradient also improves the tail of the response function. This shows that the gradient design, which was originally discussed primarily in the context of 10 meV-resolution analyzers with large solid angle (∼50 mrad × 50 mrad), may also be used for high-resolution analyzers. Work is continuing on the 10 meV setup.

## Figures and Tables

**Table 1:** Resolution for the BL43LXU spectrometer as calculated *via* ray-tracing Resolutions given are FWHM. *E*: photon energy; *d*: sample–detector vertical offset; δ_0_: deviation angle from exact backscattering at the center of the analyzer; Δδ (= δ_max_ − δ_min_): Bragg angle distribution over the analyzer; (Δ*E*/*E*)_geom2_ ≡ Δδtanδ_0_: fractional energy resolution by demagnification contribution; (Δ*E*/*E*)_geom1_ ≡ (*c*/*L*_1_)tanδ_0_: fractional energy resolution by crystallite size contribution; Δ*E*_geom_: geometrical broadening; Δ*E*_tot_: theoretical total energy resolution; 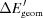, 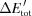: Δ*E*_geom_ and Δ*E*_tot_ with temperature gradient; Δ*T*_g_: temperature gradient to reduce demagnification contribution. Here, radius of curvature: *R* = 9800 mm; sample–analyzer distance: *L*_1_ = 9926.6 mm; analyzer–detector distance: *L*_2_ = 9676.6 mm; sample–detector horizontal offset: *l* = 250 mm.

					Uniform temperature			Temperature gradient			
Case	*E* (keV)	*d* (mm)	δ_0_ (mrad)	Δδ (mrad)	(Δ*E*/*E*)_geom2_ (×10^−8^)	Δ*E*_geom_ (meV)	Δ*E*_tot_ (meV)	(Δ*E*/*E*)_geom1_ (×10^−8^)	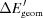 (meV)	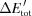 (meV)	Δ*T*_g_ (mK/80 mm)
1	25.702	4	0.204	0.112	2.29	0.57	0.81	1.79	0.43	0.65	8.08
2	25.702	10	0.510	0.112	5.71	1.46	1.64	4.47	1.06	1.21	20.2
3	21.747	4	0.204	0.112	2.29	0.48	1.27	1.79	0.37	1.21	8.08
4	21.747	10	0.510	0.112	5.71	1.22	1.74	4.47	0.96	1.42	20.2

**Table 2:** Offset heater output for two focusing geometries at BL43LXU Parentheses indicate calculated minimum values. *P*: applied power for offset heaters; *V*_*f*_: measured applied voltage for offset heaters.

		Measurement			Calculation	
Case	*d* (mm)	Δ*T*_g_ (mK/80 mm)	*P* (mW)	*V*_*f*_ (V)	Δ*T*_g_ (mK/80 mm)	*P* (mW)
1	4	10	58.7	1.6–1.7	8.1	47
2	10	25	155.9	2.6–2.8	20.2	118

**Table 3:** Measured total energy resolution width with temperature gradient at BL43LXU Parentheses indicate uniform temperature values. The theoretical limits with the temperature gradient are also listed. FWM/10: full-width at tenth of maximum; FWM/100: full width at one-hundredth of maximum.

			Calculation	Measurement		
Case	*E* (keV)	*d* (mm)	FWHM (meV)	FWHM (meV)	FWM/10 (meV)	FWM/100 (meV)
1	25.702	4	0.65	0.75 (0.96)	1.9 (2.2)	7.4 (8.0)
2	25.702	10	1.21	1.31 (1.92)	2.8 (3.7)	9.9 (11.9)
3	21.747	4	1.21	1.25 (1.50)	3.1 (3.6)	10.9 (12.2)
4	21.747	10	1.42	1.55 (1.97)	4.0 (4.5)	12.6 (14.2)

## Footnotes

1 The present spectrometer has flexibility to operate between on-Rowland and off-Rowland geometry. The sample–analyzer distance *L*_1_ is adjustable from 9800 to 10100 mm.

2 ±0.3 mK corresponds to ±0.017 meV for 21.7 keV and ±0.02 meV for 25.7 keV.

## References

[bb1] Baron, A. Q. R. (2010). *SPring-8 Inf. Newsl.* **15**, 14.

[bb2] Baron, A. Q. R., Sutter, J. P., Tsutsui, S., Uchiyama, H., Masui, T., Tajima, S., Heid, R. & Bohnen, K.-P. (2008). *J. Phys. Chem. Solids*, **69**, 3100–3102.

[bb3] Baron, A. Q. R., Tanaka, Y., Goto, S., Takeshita, K., Matsushita, T. & Ishikawa, T. (2000). *J. Phys. Chem. Solids*, **61**, 461–465.

[bb4] Bechhoefer, J. (2005). *Rev. Mod. Phys.* **77**, 783–836.

[bb5] Burkel, E. (1991). *Inelastic Scattering of X-rays with High Energy Resolution, Springer Tracts in Modern Physics*, Vol. 125. Berlin: Springer.

[bb6] Dorner, D. & Peisl, J. (1983). *Nucl. Instrum. Methods Phys. Res.*, **208**, 587–592.

[bb8] Fujii, Y., Hastings, J., Ulc, S. & Monctonl, D. (1982). Technical Report VIII-95. SSRL, Stanford, CA, USA.

[bb9] Fukui, H., Katsura, T., Kuribayashi, T., Matsuzaki, T., Yoneda, A., Ito, E., Kudoh, Y., Tsutsui, S. & Baron, A. Q. R. (2008). *J. Synchrotron Rad.* **15**, 618–623. 10.1107/S0909049508023248 18955769

[bb10] Glassbrenner, C. & Slack, G. (1964). *Phys. Rev.* **134**, A1058.

[bb11] Huotari, S., Albergamo, F., Vankó, G., Verbeni, R. & Monaco, G. J. (2006). *Rev. Sci. Instrum.* **77**, 053102.

[bb12] Huotari, S., Vankó, Gy., Albergamo, F., Ponchut, C., Graafsma, H., Henriquet, C., Verbeni, R. & Monaco, G. (2005). *J. Synchrotron Rad.* **12**, 467–472. 10.1107/S0909049505010630 15968123

[bb13] Ishikawa, D. & Baron, A. Q. R. (2010). *J. Synchrotron Rad.* **17**, 12–24. 10.1107/S0909049509043167 PMC2797303 20029107

[bb14] Masciovecchio, C., Bergmann, U., Krisch, M., Ruocco, G., Sette, F. & Verbeni, R. (1996*a*). *Nucl. Instrum. Methods Phys. Res. B*, **111**, 181–186.

[bb15] Masciovecchio, C., Bergmann, U., Krisch, M., Ruocco, G., Sette, F. & Verbeni, R. (1996*b*). *Nucl. Instrum. Methods Phys. Res. B*, **111**, 339–340.

[bb16] Miwa, D. (2002). Master’s thesis, Himeji Institute of Technology, Japan.

[bb18] Said, A. H., Sinn, H. & Divan, R. (2011). *J. Synchrotron Rad.* **18**, 492–496. 10.1107/S0909049511001828 PMC3268696 21525659

[bb19] Sette, F., Krisch, M. H., Masciovecchio, C., Ruocco, G. & Monaco, G. (1998). *Science*, **280**, 1550–1555.

[bb20] Sinn, H., Alp, E. E., Alatas, A., Barraza, J., Bortel, G., Burkel, E., Shu, D., Sturhahn, W., Sutter, J. P., Toellner, T. S. & Zhao, J. (2001). *Nucl. Instrum. Methods Phys. Res. A*, **467**–**468**, 1545–1548.

[bb21] Verbeni, R., Kocsis, M., Huotari, S., Krisch, M., Monaco, G., Sette, F. & Vanko, G. (2005). *J. Phys. Chem. Solids*, **66**, 2299–2305.

